# Indocyanine Green Nanoparticles: Are They Compelling for Cancer Treatment?

**DOI:** 10.3389/fchem.2020.00535

**Published:** 2020-07-16

**Authors:** Marta Sevieri, Filippo Silva, Arianna Bonizzi, Leopoldo Sitia, Marta Truffi, Serena Mazzucchelli, Fabio Corsi

**Affiliations:** ^1^Laboratorio di Nanomedicina, Dipartimento di Scienze Biomediche e Cliniche “L. Sacco”, Università di Milano, Milan, Italy; ^2^Laboratorio di Nanomedicina e Imaging Molecolare, Istituti Clinici Scientifici Spa-Società Benefit IRCCS, Pavia, Italy

**Keywords:** nanoparticles, cancer, indocyanine green (ICG), diagnosis, treatment

## Abstract

Indocyanine green (ICG) is a Food and Drug Administration–approved near-infrared fluorescent dye, employed as an imaging agent for different clinical applications due to its attractive physicochemical properties, high sensitivity, and safety. However, free ICG suffers from some drawbacks, such as relatively short circulation half-life, concentration-dependent aggregation, and rapid clearance from the body, which would confine its feasible application in oncology. Here, we aim to discuss encapsulation of ICG within a nanoparticle formulation as a strategy to overcome some of its current limitations and to enlarge its possible applications in cancer diagnosis and treatment. Our purpose is to provide a short but exhaustive overview of clinical outcomes that these nanocomposites would provide, discussing opportunities, limitations, and possible impacts with regard to the main clinical needs in oncology.

## Introduction

Recently, growing attention has been addressed to nanocarriers for Indocyanine green (ICG) delivery with the purpose of overcoming some of its current limitations and to expand its possible applications in cancer diagnosis and treatment (Wang et al., 2018). ICG is a widely investigated near infrared (NIR) fluorescent agent, approved for clinical use by the Food and Drug Administration (FDA) in the 1950s (Landsman et al., 1976; Alius et al., 2018). Over the past decade, NIR optical imaging using ICG has become determining for a variety of applications, including lymphangiography, intra-operative lymph node (LN) identification, tissue perfusion, detection of vital structures, and tumor imaging (Fox and Wood, 1960; Starosolski et al., 2017). ICG displays several advantages thoroughly verified during its long clinical use: it is easy to use, cost-effective, radiation-free, and safe. Although ICG fluorescent imaging represents a promising medical tool, its application remains limited due to the intrinsic issues related to ICG degradation and rapid blood clearance (Muckle, 1976; Saxena et al., 2003; Zheng et al., 2012). Therefore, many studies suggest that the exploitation of ICG-based nano-formulations (micelles, polymeric nanoparticles, silica nanoparticles, and liposomes) could boost the efficacy, specificity, and biosafety of this imaging agent for potential oncological applications (Yan et al., 2016; Egloff-Juras et al., 2019).

## Properties of ICG

ICG is an amphiphilic tricarbocyanine dye used in the biomedical field for almost six decades for different purposes (Schaafsma et al., 2011; Hill et al., 2015). ICG is an anionic, water-soluble, and fluorescent molecule with a molecular weight of 751 Da and that displays absorption and fluorescence emission in the NIR wavelength region (Zhao et al., 2014). These properties allow deep penetration of the signal and minimize interference of tissues' autofluorescence, making it suitable for bio-imaging uses (Wang et al., 2004; Yuan et al., 2004). Moreover, since it is an FDA-approved dye, well studied in its already known clinical applications, its introduction to new clinical applications is greatly simplified (Alander et al., 2012; Valente et al., 2019). Additionally, due to its photosensitizing properties, ICG can be used to generate oxygen species (ROS) or heat, aiming to destroy cancer cells in photodynamic therapy (PDT) and photothermal therapy (PTT) (Dolmans et al., 2003; Kuo et al., 2012). Despite these compelling properties, the application of ICG is restricted due to its concentration-dependent aggregation, quick degradation, and poor photostability. Furthermore, its non-specific binding to plasma proteins determines a relatively short circulation half-life, and its non-specific targeting remains a limitation (Kirchherr et al., 2009; Yaseen et al., 2009).

## ICG as an NIR Fluorescent Contrast Agent: Clinical Applications

As previously mentioned, ICG has an excellent safety profile and, following injection of a clinical standard dose (0.1–0.5 mg/kg), immediately interacts with plasma proteins, acting as an excellent vascular agent for evaluating both the blood perfusion and lymphatic drainage (Alford et al., 2009; Marshall et al., 2010; Boni et al., 2015). Once excited at the wavelength of about 820 nm, ICG emits a fluorescent signal detectable by specific scopes and cameras to allow identification of anatomical structures where the dye localizes (Luo et al., 2011; Daskalaki et al., 2014). Indeed, ICG is used in intraoperative angiography for assessment of superficial eye vessels and in the evaluation of coronary artery bypass grafts, peripheral vascular disease, and solid organ transplantation (Reuthebuch et al., 2004; Sekijima et al., 2004; Desai et al., 2006; Kang et al., 2010a,b; Baillif et al., 2011). Moreover, since, once injected intravenously, ICG is excreted exclusively via the liver, it is used to assess hepatic function (Daskalaki et al., 2014). In addition to these applications, ICG is employed in NIR fluorescence image-guided oncologic surgery with the purpose of identifying structures that need to be resected (e.g., tumor tissue, lymph nodes) and spared, contributing to support the surgeon's decision-making process (Boni et al., 2015; Baiocchi et al., 2018).

Of note, NIR fluorescence imaging via ICG can provide real-time identification of tumor margins and affected lymph nodes (LN) in breast and skin cancers, improving local control of the disease and allowing a more conservative surgery (Sevick-Muraca et al., 2008; Fujiwara et al., 2009; Murawa et al., 2009). Sentinel LN (SLN) mapping is important to detect involved LN and is required for cancer staging, prognosis prediction, and therapy selection (Schaafsma et al., 2011; Wang et al., 2018). Here, ICG is injected near the tumor and flows via lymph circulation to LN, displaying them when lit with excitation light (Tanaka et al., 2006; Alander et al., 2012; Wishart et al., 2012; Verbeek et al., 2014). ICG-NIR fluorescence imaging has been applied also to intraoperative tumor detection in order to ensure a total tumor resection (Gotoh et al., 2009; Onda et al., 2016; Rossi et al., 2018). Indeed, exploiting ICG hepatic clearance and the enhanced permeability and retention (EPR) effect, liver tumors could be identified (Ishizawa et al., 2009; Huang et al., 2018).

To date, the application of ICG as an NIR fluorescence imaging agent in oncology is an active and promising area, but it also has limitations. Aside from the problems inherent with some of its physicochemical properties, ICG is a non-targeted or extremely low targeted tracer, which greatly precludes its application for specific cancer imaging (Landsman et al., 1976; Marshall et al., 2010; Wang et al., 2018; Egloff-Juras et al., 2019).

## ICG-NPs and Cancer: Preclinical Studies

**Figure 1:**
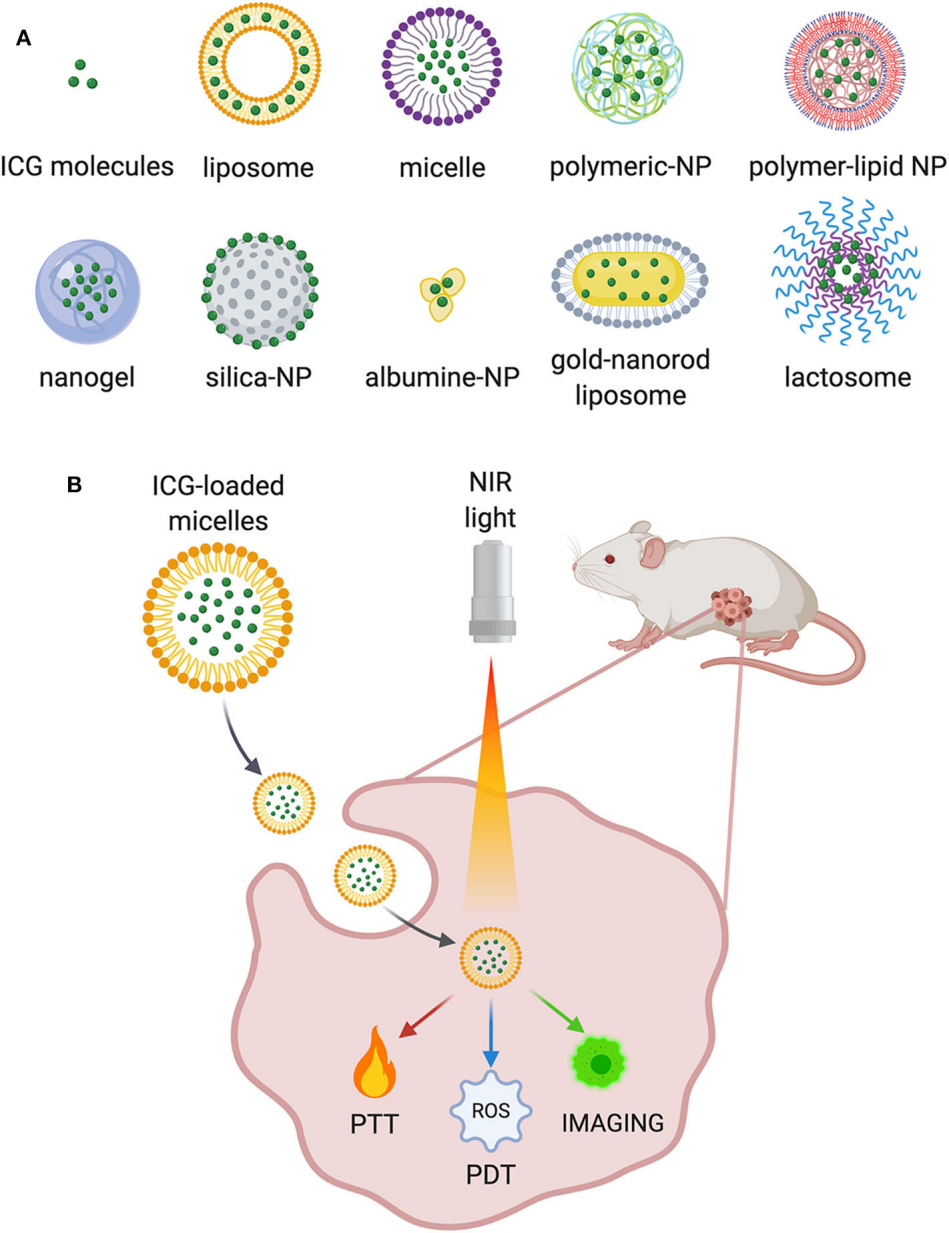
**(A)** Some examples of the ICG-NPs obtained with different materials and conjugation techniques. **(B)** Main applications of ICG-NPs in cancer treatment.

Recently, the development of multifunctional ICG-NPs, offering both diagnostic and therapeutic solutions in cancer, has captured the attention of researchers (Han et al., 2018). To overcome the limitations previously discussed, several ICG-NPs have been proposed and tested, both *in vitro* and *in vivo* (Liu et al., 2019b; ZhuGe et al., 2019), displaying increased circulation time and improved ICG optical properties and achieving tumor-specific accumulation. Many advantages derive from their use: combining or encapsulating it to/into NPs results in the extension of ICG half-life. Additionally, functionalization with specific cancer-related antibodies may result in preferential accumulation of ICG at the tumor site. Furthermore, ICG-NPs may be useful to limit ICG aggregation and photodegradation as well as to improve its stability in aqueous solutions (Ishizawa et al., 2009; Liu et al., 2019c; ZhuGe et al., 2019). ICG has been loaded or conjugated to a variety of nanostructures, such as polymer-based NPs, lipid-based NPs, and silica NPs with different surface modifications and functionalization strategies (Figure 1A). Among the plethora of ICG-NPs for different targets and applications, we focused on the following main applications for cancer treatment: PDT and PTT (i), *in vivo* imaging and image-guided surgery (ii), and multimodal therapy (iii) (Figure 1B). A summary of all significant examples of ICG-NPs developed for these applications has been inserted in Table 1.

**Table 1:** Summary of all significant examples of ICG nanoparticles studied *in vivo*.

**ICG-NPs: preclinical research**					
**Nanoparticle type**	**Conjugation technique**	**Target**	**Aim/use**	**Achievements/findings**	**References**
Calcium phosphate nanoparticles	ICG encapsulation	Breast cancer	Fluoroprobing	Improved half life Improved photostability Biocompatibility	Altinoglu et al., 2008
Water-responsive phospholipid-calcium-carbonate hybrid nanoparticle	ICG loading doxorubicin loading	Breast cancer	PTT	Good tissue penetration Decreased metastatic areas	Liu et al., 2019b
Super carbonate apatite nanoparticles	ICG loading	Colorectal adenocarcinoma	PDT	Fluorescence enhancement Significant tumor growth retardation	Tamai et al., 2018
Hydrophobic superparamagnetic iron oxide nanoparticles	ICG loading doxorubicin coating	Glioblastoma	MRI imaging Chemotherapy	BBB crossing Accumulation at the tumor site	Shen et al., 2018
Graphene oxide hybrid nano-composites	Electrostatic interaction with ICG	Colorectal adenocarcinoma	PTT	Citotoxicity in cancer cells only	Sharker et al., 2015
Polyamidoamine (PAMAM)-coated silica nanoparticles	ICG coating	Sentinel lymph nodes	Sentinel lymph node imaging	Facilitatation of sentinel lymph node biopsy procedures	Tsuchimochi et al., 2013
PL-PEG-mAb nanoparticles	ICG-PEG conjugation	Glioblastoma Breast cancer	PTT Imaging	Good targeting Tumor reduction	Zheng et al., 2012
PLGA-PEG-R837 nanoparticles	ICG encapsulation	Breast cancer	PTT Immunotherapy	Great antitumor effect Strong immune-memory effect	Chen et al., 2016
PEG-PCL-C3 hybrid nanoparticles	ICG-PEG conjugation	Oral squamous cell carcinoma	PTT PDT	*In vivo* safety Active role in reducing tumor volume	Ren et al., 2017
Levan nanoparticles	ICG encapsulation	Breast cancer	Imaging	Selective targeting of cancer cells	Kim et al., 2015
Silk fibroin nanoparticles cross-linked by proanthocyanidins	ICG encapsulation	Glioblastoma	PTT	Stable photothermal properties Decrease of tumor volume	ZhuGe et al., 2019
Silk fibroin nanoparticles	ICG encapsulation	Glioblastoma	PTT	Inhibition of tumor growth	Xu et al., 2018
Hyaluronic acid nanoformulation	ICG entrapment	Pancreatic cancer	Tumor detection	Safe contrast agent	Qi et al., 2018
Hyaluronic acid nanoformulation	ICG entrapment	Breast cancer	Image-guided surgery	Good contrast enhancement	Hill et al., 2015
Hyaluronic acid nanogels	ICG entrapment	Breast cancer	Imaging	Improved imaging of metastatic lymph nodes	Mok et al., 2012
Polymer-lipid nanoparticles	ICG encapsulation	Pancreatic cancer	PTT	Suppression of tumor growth	Zhao et al., 2014
Mannosylated liposomes	ICG encapsulation	Sentinel lymph nodes	Sentinel lymph node imaging	Increased liposomal stability Good optical properties	Jeong et al., 2013
Liposomes	Lipid-bound ICG	Healthy organism	Imaging	Fluorescence enhancement	Kraft and Ho, 2014
Liposomes	ICG-iDOPE incorporation	Glioblastoma	PDT	Suppression of tumor growth	Shibata et al., 2019
Gold nanorod@liposome core–shell nanoparticles	ICG loading	Liver cancer	Photoacoustic tomography Surgery Guidance	Prolonged half-life Preoperative detection of liver cancer	Guan et al., 2017
Micelles	ICG-PEG conjugation	Lung carcinoma	PTT	Tumor detection Inhibition of tumor growth	Li et al., 2019b
Micelles	ICG/retinal loading	Murine breast cancer	PTT	Suppression of tumor growth	Zhu et al., 2019
Lactosomes (micelles assembled from block copolymers)	ICG loading	Metastatic lymph nodes in gastric cancer	PDT Imaging	Selective accumulation in metastatic lymph nodes	Tsujimoto et al., 2015
^*^Phospholipid nanoprobes Folic acid-phospholipid nanoparticles	ICG-PEG conjugation	Glioblastoma Breast cancer	PTT Imaging	Selective imaging of cancer cells Selective killing of cancer cells	Zheng et al., 2011
Folate-targeted lipid nanoparticles	ICG/oxygen loading	Ovarian cancer	PTT PDT Imaging	Good targeting Increased PDT efficacy	Liu et al., 2019c
Folic acid-targeted nanoparticles	ICG loading	Breast cancer	PTT	Significant targeting to MCF-7 tumors Tumor growth inhibition	Zheng et al., 2014
HDL nanoparticles	ICG encapsulation	Murine breast cancer	PTT PDT	Deep tumor penetration Enhanced tumor necrosis	Sheng et al., 2019
Human ferritin	Photosensitizer encapsulation	Glioblastoma	PDT	High phototoxicity in tumors Normal tissue left unaffected	Zhen et al., 2013
BSA nanoparticles	ICG coating artemisin encapsulation	Epidermal carcinoma	PTT PDT	Synergistic photo-chemotherapy	Ma et al., 2018
Human serum albumin	ICG adsorption	Murine breast cancer	PTT PDT	Tumor margin detenction Tumor eradication without regrowth	Sheng et al., 2014
^§^Human serum albumin	ICG adsorption	Breast cancer	Sentinel lymph node mapping	Clinical trial	Hutteman et al., 2011

* *in vitro research only*.

§ *clinical trial*.

PDT is an emerging, minimally invasive cancer treatment based on the production of ROS in response to a source of light, the presence of oxygen and a photosensitizer (i.e., ICG) (ZhuGe et al., 2019). Although this option seems promising for many cancers (Gross et al., 2003; Ritch and Punnen, 2017), selective delivery of the photosensitizers at target tissues/cells remains insufficient for successful clinical use (Zhen et al., 2013). Since PDT could generate an antitumor immune response, ICG-loaded liposomes were studied in combination with NIR irradiation, demonstrating suppression of brain tumor growth and suggesting the potential application for the treatment of tumors near the brain surface (Shibata et al., 2019). Super carbonate apatite-ICG NPs improved ICG uptake in tumor cells and its antitumor effect in a colorectal xenograft model, serving as a useful vehicle for ICG-based PDT (Tamai et al., 2018). Also, hydroxyethyl starch-oleic acid ICG-NPs exhibited excellent stability and efficient ROS generation and increased cellular uptake and tumor accumulation compared to free ICG (Hu et al., 2019).

In addition to PDT, PTT also arose as a promising approach for cancer treatment by using NIR-light to generate heat and achieve tumor ablation (Li et al., 2019a). The main challenge with PTT is that heat could also damage the healthy surrounding tissue and fail to eradicate metastatic cells. Several NPs with excellent NIR light absorption have been developed as PTT agents, including gold, copper, carbon NPs, and NIR dyes (Lv et al., 2017). Also, ICG has been exploited in a multitude of NPs as a PTT agent (Doughty et al., 2019). ICG-conjugated micelles have been investigated for breast and lung cancer treatment displaying increased circulation time, accurate tumor targeting, and efficient PTT effect compared with free ICG (Li et al., 2019b; Zhu et al., 2019). Another work proposed the functionalization with folic acid to achieve accumulation on MCF-7 breast cancer cells, obtaining a significant tumor growth inhibition (Zheng et al., 2014). A formulation of pH-responsive polymeric nano-complexes of graphene oxide and ICG (Sharker et al., 2015) was effective in providing selective sensitivity to tumor environment and tumor regression, confirming its clinical usefulness. Also, in studies with ICG-loaded polymer-lipid NPs against pancreatic cancer (Zhao et al., 2014) and silk fibroin NPs addressing glioblastoma (Xu et al., 2018), the main advantages observed, compared to free ICG, were an extended circulation time and *in vivo* stability, together with the ability to specifically target cancer cells (Sheng et al., 2019).

An assortment of ICG-NPs also has been developed for bioimaging applications as agents for tumor identification. Since early detection is crucial for the prompt diagnosis and successful treatment of cancer, the benefits of using NPs as vector for ICG to the tumor site would be significant. ICG-incorporating liposomes provide enhanced visualization of the popliteal LN and downstream LN, detected across 1.5 cm of muscle tissue, and free ICG only enables 0.5 cm detection (Kraft and Ho, 2014). Hyaluronic acid (HA) NPs allow contrast enhancement (Hill et al., 2015), and levan NPs display good targeted imaging of breast tumors and the suitability to encapsulate hydrophobic drugs (Kim et al., 2015). ICG-HA–derived NPs improve the NIR signal for intraoperative detection of pancreas and splenic metastasis compared to ICG (Qi et al., 2018), and also nanogels display good performance in targeted imaging of cancers and LN metastases in addition to the feasible drug-encapsulation in their hydrophobic core (Mok et al., 2012). ICG-doped calcium phosphate NPs display increased deep-tissue penetration (Altinoglu et al., 2008). Another promising strategy involves its effectiveness as a photoacoustic-fluorescence imaging probe in liver cancer detection (Guan et al., 2017). Overall, all the considered formulations display a non-toxic safety profile, a longer circulation time, and a higher tumor accumulation than free ICG. This would provide the potential to increase the completeness of surgery and the chances of a better outcome. Furthermore, different ICG-NPs were suggested as contrast agents for SLN mapping. Mannosilated ICG-liposomes show improved stability and fluorescence signal by exploiting their specific recognition by macrophages, making it a good agent for SLN and LN imaging (Jeong et al., 2013). Silica NPs loaded with technetium and ICG improve LN detection in real time although further studies are necessary to assess the appropriate dose (Tsuchimochi et al., 2013), and ICG-loaded lactosomes provide an improved LN detection and a inhibited growth upon PDT treatment (Tsujimoto et al., 2015). The only example tested in the clinic concerns the use of ICG adsorbed to human serum albumin (ICG:HSA) aiming to improve detection and better retention in the SLN after intradermal injection. However, this trial performed on breast cancer patients showed no advantage of ICG:HSA for SLN mapping (Hutteman et al., 2011).

Very often, the applications described above have been used in combination to obtain better therapeutic results. Indeed, many authors consider the use of ICG for imaging-guided PTT, allowing simultaneously tumor detection and eradication. ICG-PL-PEG NPs were investigated *in vitro* for cell imaging and selective PTT, proving to be an interesting multifunctional system (Zheng et al., 2011), and Liu and coworkers provide a synergistic strategy for both offering contrast enhancement and tumor growth reduction against ovarian cancer (Liu et al., 2019c). Furthermore, the successful application of HSA-ICG NPs for *in vivo* imaging and tumor margin detection following PDT/PTT synergic phototherapy has been reported (Sheng et al., 2014). Many authors also insist on the strength of the synergistic combination of PTT and PDT to obtain better therapeutic results (Ren et al., 2017; Sheng et al., 2019; Zhu et al., 2019).

Moreover, since monotherapy, either PTT or PDT usually suffers from incomplete tumor killing, leading potentially to tumor relapse (Ma et al., 2018); a combination with chemotherapy could optimize the cancer treatment. In this context, chemotherapy drugs combined with phototherapy have been studied. Doxorubicin has been exploited in superparamagnetic iron oxide NPs with ICG displaying good imaging ability, showing accumulation in the tumor site and high antitumor efficacy with few side effects in glioma-bearing rats (Shen et al., 2018). Phospholipid-calcium-carbonate NPs loaded with doxorubicin and ICG demonstrate strong tumor-homing properties and a synergistic effect in terms of tumor growth reduction (Liu et al., 2019b). Regarding cancer immunotherapy, the photothermal ablation of tumors with immune-adjuvant ICG-NPs, seems promising in activating immune responses potentially applicable for metastasis treatment (Chen et al., 2016). Overall, multimodal therapies appear to enhance the therapeutic effects and prevent possible recurrences.

## ICG-NPs: Opportunities, Limitations and Possible Impacts in Oncology

Implementing a plethora of different ICG-NPs makes the comparison between them especially difficult. Overall, the most promising strategies are related to actively targeted ICG-NPs, but there is a significant gap in outcomes between preclinical cancer models and their translation into clinical practice. As previously discussed, several recent studies are focusing on the development of ICG-NPs, aiming to exploit the advantages of ICG in order to further increase cancer therapies.

First, ICG-NPs could be effective in improving the already existing imaging techniques, either by prolonging ICG half-life or by selectively addressing the molecule to cancer cells only; that could be particularly relevant when trying to identify metastases as well as being useful to early detection of cancer cells in case of relapse. The chance to use NPs-ICG as drugs to directly treat tumors represents an additional advantage. By exploiting the ability of ICG to both generate heat and ROS in response to NIR, ICG-NPs could be used for PTT, PDT, or both in order to elicit antitumor response (Han et al., 2018; Liu et al., 2019c). Indeed, the accumulation of ICG in tumor cells and their exposure to light, determines a localized increase in temperature that causes cell damage by apoptosis and necrosis, resulting in tumor ablation (Melamed et al., 2015; Pérez-Hernández et al., 2015). In the meantime, in the presence of oxygen, light-activated ICG also generates ROS, leading to cell death and tissue destruction (Allison and Moghissi, 2013). Nevertheless, recent studies are quite misleading about the application of PTT and PDT, which rely on different therapeutic mechanisms. However, when ICG is used, both effects could be achieved although with a distinct tumor cell–killing contribution by each (Liu et al., 2019a). More clarity about which strategy is being referred to is necessary since, through ICG, one thing does not exclude the other, and the related side effects should be considered as well. Both PTT and PDT are promising for the treatment of several malignancies; however, it is important to understand which aspect to target to design the appropriate NPs (Pinto and Pocard, 2018).

The first concern is about immunogenicity. Although it is common opinion that ICG itself is not toxic and that ICG-NPs can be selectively targeted to the tumor, it is still uncertain if other tissues could be affected by the treatment. NPs must not elicit an immune response and demonstrate not to be toxic for the organism.

A second issue is obvious: cancer is not a single disease. Tumors may be solid or not, have clear or irregular borders, spread in easily reachable districts or in hard-to-treat areas. Additionally, similar tumors may have different density, vasculature, and tumor microenvironment. Here, the ability of ICG-NPs to penetrate into the tumor is unclear, thus making it hard to decide if ICG-NPs should be addressed to primary tumors and metastases or used as adjuvants after surgery (Tsujimoto et al., 2015; Sheng et al., 2019). Research on ICG-NPs should develop the optimal strategy for real clinical applications on each disease instead of just developing NPs that prove to be effective at a preclinical stage but are difficult to translate into clinical trials.

Regarding the multitude of developed ICG-NPs, one fact is evident: every group focuses on the NP type they are used to working with as well as on the tumor model they know better (Table 1). Such an approach is both useful and harmful. On the one hand, long-term expertise in developing a specific NP could be addressed to the production of highly effective ICG-NPs; on the other hand, several cancer models should be tested to evaluate the efficacy of the proposed treatment in different contexts. Focusing on a single model could be limiting if the purpose is to design a consistent model for the development of new therapies. Options to design ICG-NPs are unlimited, and many could be adjusted to target different tumors (Han et al., 2018). NPs can be further enriched by conjugation with other molecules: monoclonal antibodies, fluorescence probes and drugs in order to maximize the antitumor effect of the ICG-NPs (Zheng et al., 2011; Sheng et al., 2014; Ma et al., 2018). Here, given that the enhancement of targeting is crucial to prevent damage of healthy tissues, NPs should be selectively directed to the tumor, either inserting targeting molecules or by exploiting the intrinsic ability of some carriers to bind cancer.

Despite all the advantages deriving from ICG-NPs and the huge amount of solutions proposed so far, a pool of clinical applications have not been outlined yet, thus making it impossible to determine which ones have the potential to become actual drugs. The answer is not one and only: different nanoparticles could be used to treat different tumors, and different molecules could be attached to improve their selectivity. Therefore, developing dozens of different ICG-NPs is not of help in finding the best alternatives that could be eventually tested in clinical trials.

On top of everything, when discussing the potentiality of ICG-NPs in therapy, an uncomfortable yet necessary question should be raised about the costs of clinical trials involving NPs: developing such molecules, especially when they are combined with chemotherapeutic drugs and/or patented monoclonal antibodies, has been proved to be extremely expensive, thus limiting the possibility to produce high amounts of molecules to be used in clinical trials.

## Discussion

In conclusion, the potential of ICG-conjugated NPs is undeniable, mostly because they could possibly be directed toward several cancer types with incredibly high specificity (Bozkulak et al., 2009; Montazerabadi et al., 2012). Indeed, they could overcome some of the limitations of current treatments, especially regarding tumors that are poorly accessible by drugs or hard to treat, and they could also limit the side effects usually associated to conventional therapies (Montazerabadi et al., 2012). However, obtaining specific cell targeting as well as maintaining high drug concentration at the tumor site remain the main challenges as they are both necessary conditions for the implementation of ICG-NPs–based imaging, PTT, and PDT. This is the reason why the currently known ICG-NPs have not successfully reached the translation into clinics so far. Therefore, an improved active targeting is required for a major impact on human health. Moreover, since the penetration depth of light in tissues could be limited, even with NIR lasers, endoscope-based clinical devices equipped with a laser may be demanded in order to reach successful outcomes in clinical practice (Chen et al., 2016). Some sort of consensus should also be achieved about the most promising formulations and the real aims of the proposed interventions: PTT, PDT, and imaging are not interchangeable terms, and more precision is required when deciding which therapies would be worth testing. In addition, before trying to develop new ICG-NPs, the economic impact of a potential trial involving such NPs must be carefully considered. Expertise and deep knowledge in the field are mandatory, but feasibility will eventually determine if a promising molecule will ever be translated into an actual therapy.

## Author Contributions

MS, FS, AB, and LS contributed in manuscript writing and figure preparation. SM, MT, and FC contributed in manuscript writing, editing, and critical review. All authors contributed to the article and approved the submitted version.

## Conflict of Interest

The authors declare that the research was conducted in the absence of any commercial or financial relationships that could be construed as a potential conflict of interest.
